# The genetic structuring in pollinating wasps of *Ficus hispida* in continental Asia

**DOI:** 10.1002/ece3.10518

**Published:** 2023-09-20

**Authors:** Xiaoxia Deng, Yaolin Liao, Da‐Mien Wong, Hui Yu

**Affiliations:** ^1^ Key Laboratory of Plant Resource Conservation and Sustainable Utilization South China Botanical Garden, CAS Guangzhou China; ^2^ South China National Botanical Garden Guangzhou China; ^3^ CEFE CNRS, Univ Montpellier, EPHE, IRD Montpellier France

**Keywords:** co‐pollinators, *Ficus*, fig‐pollinating wasp, host specificity

## Abstract

The interaction between figs and fig wasps provides a striking example of obligate brood site pollination mutualism. Monoecious figs, constituting independent radiations in each tropical biome, are present in significant proportions worldwide, but in continental Asia, dioecious figs have diverged into various niches, making the region's assemblage remarkably diverse. However, the reproductive success of figs and fig wasps largely depends on the fig wasp dispersal process. Monoecious fig pollinators in continental Asian tropical rain forests exhibit high gene flow of the plant, while many dioecious fig pollinators have a more restricted gene flow. However, there are limited studies on the genetic structure of dioecious *Ficus* pollinators that pollinate figs with intermediate gene flow. Here, we used molecular methods to investigate the genetic structure of pollinating wasps of the widely distributed dioecious *Ficus hispida* in China and Southeast Asia. Sequence data from two gene regions were used: the mitochondrial protein‐coding gene cytochrome c oxidase subunit I (COI) and the nuclear 28S genes. Both molecular and morphological results support two fig wasp species at our sampling sites. Our findings suggest that for widely sympatric *Ficus* species in continental Asia, monoecious figs presenting long gene glow have the fewest fig wasp species, followed by dioecious figs presenting intermediate gene flow, and dioecious figs presenting local gene flow have the most fig wasp species.

## Graphical abstract

In the Asian flora, there are usually more dioecious *Ficus* species than monoecious ones. However, the reproductive success of figs and fig wasps largely depends on the fig wasp dispersal process. Population structuring by colonization of new niches could be the most important mechanism of species radiation in continental areas.

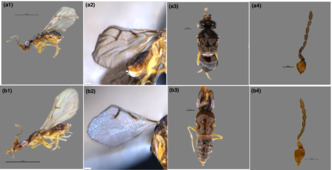

## 1 INTRODUCTION

Pollination is the process whereby pollen is transferred from anthers (male part) and deposited onto the stigma(s) (female part) of flowers resulting in fertilization and the production of seeds (sexual reproduction; Sakai, 2002). The pollination mutualism between plants and their pollinators has played a major role in the maintenance of terrestrial biodiversity (Bascompte et al., 2006; Benton et al., 2022; Carvalho et al., 2021). Brood‐site pollination mutualisms in which plants provide pollinators with sites for offspring development or mimic the presence of these sites in exchange for pollination services represent extreme levels of reciprocal specialization between plants and insect pollinators (Borges, 2016; Sakai, 2002; Suinyuy et al., 2015). In such mutualisms, pollinator species use one or a few plant species as hosts, and each plant species is pollinated by one or a few insect species (Cousins & Witkowski, 2017; Dufaÿ et al., 2003; Kawakita & Kato, 2009; Okamoto et al., 2013; Shi et al., 2019; Toon et al., 2020; Willmott et al., 2017), thus pollen‐mediated gene flow depends on the behavior of pollinators when moving pollen, as well as the spatial distribution of the plants (Alamo‐Herrera et al., 2022; Barrett, 2003; Cresswell et al., 2002; Ennos, 1994). One of the most tightly integrated pollination mutualisms known occurs in the obligate relationship between figs (*Ficus* spp.) and their pollinating wasps (Agaonidae, Chalcidoidea; Harrison et al., 2008; Herre et al., 2008).

Some 820 species of *Ficus* are distributed throughout the world's tropical and subtropical regions (Berg, 1989; Berg & Corner, 2005; Harrison, 2005). Fig trees have reciprocally obligate pollination relationships with tiny (1–2 mm), short‐lived (1–2 days adult lifespan) fig wasps (Chalcidoidea, Agaonidae). The inflorescence of figs is a closed urn‐shaped receptacle with female and male flowers inside it. Wasps oviposit into some of female flowers. Wasp larvae develop within the galled flowers and feed on the endosperm, the development of which is induced by pollination or by wasps (Jansen‐Gonzalez et al., 2012; Verkerke, 1986, 1987). Male wasps emerge first from their galls and mate with the females. Then, the adult females emerge into the fig cavity, become pollen loaded and leave and must usually fly to a different tree than her natal tree to locate receptive figs (Hossaert‐Mckey et al., 1994). Each fig species is dependent on its highly specialized fig wasp species for pollination of its flowers. In turn, fig wasps depend on figs to produce offspring and complete their life cycle. However, considering their much shorter generation lengths, fig wasps often have a diverse history of diversification from their hosts (Bain et al., 2016; Deng, Chen, et al., 2021; Rodriguez et al., 2017; Yu et al., 2019).

About half of the described species of *Ficus* are monoecious, whereas the other half are dioecious (Berg, 1989). Differences in the breeding system are associated with different traits (tree height, population density, fruiting frequency, pollinator dispersal ecology). Generally, monoecious *Ficus* are tall trees that reach the canopy, with very low population densities (<1 individual per hectare). In contrast, dioecious *Ficus* are usually small shrub‐like trees that rarely get the canopy, with high local population densities, producing fruit more frequently than monoecious trees (Harrison & Yamamura, 2003). The newly emerged pollinating wasps in monoecious figs are prone to be found above the canopy and let themselves drift in the wind until they detected the scent of their host receptive figs (Ware & Compton, 1994). Previous studies have shown that dioecious figs contribute more significantly to the spatial genetic structure of plant populations than monoecious figs (Nazareno et al., 2013) and that pollinators associated with monoecious figs dispersed more efficiently over long distances than those associated with dioecious figs (Ahmed et al., 2009; Harrison, 2003; Harrison & Rasplus, 2006; Nason et al., 1998; Zavodna et al., 2005). Furthermore, data suggested genetic co‐structuring of monoecious figs and their pollinating wasps (Bain et al., 2016), while the dioecious figs and their fig wasps usually showed discordant patterns of genetic variation and geographical divergence (Liu et al., 2013; Rodriguez et al., 2017; Yu et al., 2019).

Chemical mediation plays a crucial role in partner encounters, allowing wasps to locate host figs and find floral rewards, that is, breeding sites (Deng et al., 2022; Grison‐Pigé et al., 2002; Hossaert‐McKey et al., 2010; Proffit et al., 2007, 2009, 2020; Wang et al., 2021; Zhang et al., 2020), indicating that dispersal of pollinators and floral odors are intimately linked in the highly specialized fig‐fig wasp mutualism (Kobmoo et al., 2010; Soler et al., 2011). Previous study has shown a convergence of floral scents among *Ficus* species that share pollinators, suggesting evolutionary convergence to attract specific wasp species (Cornille et al., 2012).What do we know about within species variation in receptive inflorescence odor in fig‐fig wasp systems? The limited data suggested that in monoecious *Ficus racemose* presenting high gene flow (Bain et al., 2016), receptive fig odors do not vary from China to Thailand and odor diversification corresponds to pollinator species and plant genetics co‐structuring, as well as to climate barriers (Bain et al., 2016; Soler et al., 2011), whereas dioecious *F. hirta* presenting low gene flow (Nm = 1.7; Zheng, 2015), receptive fig odors diverge progressively among plant populations, consistent with plant genetics but not correlated with pollinator species or population distribution (Deng et al., 2022; Deng, Buatois, et al., 2021), suggesting plants are the drivers of geographic variation of floral scents and the pollinating wasps have to adapt to this variation. Earlier studies showed that in China and Southeast *F. racemosa*, a monoecious large tree, presents limited genetic differentiation and is pollinated by a single population of a single wasp species (Kobmoo et al., 2010), while *F. hirta*, a dioecious shrub or small tree, displays clinal genetic variation between locations against geographic distance (*p* < .001) and is pollinated by nine parapatric wasp species (Yu et al., 2019), revealing that the more pollinating wasp species associated with smaller *Ficus* (Chen et al., 2011; Liu et al., 2015). For the medium‐sized *F*. *hispida*, a dioecious *Ficus* species widely sympatric with *F*. *racemosa* and *F. hirta* in China and Southeast Asia, although the regression of paired *F*_ST_/(1 − *F*_ST_) versus geographic distance was also significant in *F. hispida* (*p* < .001), both published and unpublished data show significant geographic variation between China and Southeast Asia, but similar receptive fig odors in South China (Deng et al., unpublished; Soler et al., 2011), and genetic structure of *F. hispida* suggested strong south–north gene flow (Nm = 4.926) but also some east–west nuclear gene flow (Huang et al., 2023), suggesting that *F. hispida* has intermediate gene flow compared with the two *Ficus* species mentioned above. Indeed, the dispersal of pollen‐carrying female fig wasps is probably one of the most critical processes in the life cycle of fig plants and wasps. Hence, this work investigated genetic structuring in pollinating wasps of *F. hispida* from 10 populations in southern China, southwestern China, Thailand, and Indonesia. Here, we expect that the number of pollinating fig wasp species with intermediately dispersed *F. hispida* is greater than that of dispersed *F. racemosa* and smaller than that of locally dispersed *F. hirta* in continental Asia.

## 2 MATERIALS AND METHODS

**FIGURE 1:**
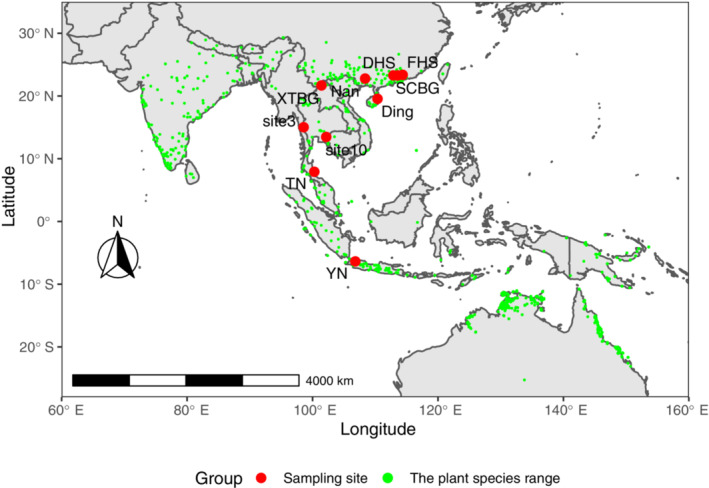
The sampling sites and the distribution of the plant species range are shown in red and green circles respectively. Six sites in China: FHS, SCBG, DHS, Nan, Ding, and XTBG. Three sites in Thailand: site3, site10, TN. One site in Indonesia: YN.

*Ficus hispida* is a pioneer species, distributed from Sri Lanka to India, South China across Southeast Asia to Australia (Figure 1), and commonly found in secondary forest and wasteland vegetation (Berg & Corner, 2005; Lee et al., 2013). Figs are produced continuously throughout the year (Corlett, 2006; Kuaraksa et al., 2012; Yang et al., 2002) and bears fruits 6–8 times, with four to five fig‐bearing peaks (Yang et al., 2002).

Between 2017 and 2020, we sampled the pollinating wasps associated with *F. hispida* from 10 locations in China (six locations), Thailand (three locations), and Indonesia (one location; Table 1 and Figure 1). Figure 1 presented both sampling site and the plant species range. The distribution data of the species were obtained from the Global Biodiversity Information Facility (GBIF) website (https://www.gbif.org/occurrence/download/0184784‐220831081235567). At each site, 3–30 near‐ripe male figs from at least two trees were collected and placed in transparent 15 × 10 cm in size, 100‐mesh bags that allowed airflow, prevented overheating, and wasp escape. In each tube, we put all the emerged wasps of each fig into 75% ethanol overnight and then transferred them to 95% ethanol and stored them at −20°C until DNA extraction. The insects were raised from naturally germinated trees found mainly at the edges of swamps and beside rivers (Berg & Corner, 2005). We used only one wasp per fig for DNA extraction in each sample.

**TABLE 1:** The geographical distribution of the different haplotypes of COI sequences.

Location	Latitude	Longitude	*N*	H	*h*	*Hd*	*Pi*
FHS	23.37	114.37	5	H1(4), H2(1)	2	0.4	0.00183
SCBG	23.28	113.58	5	H3(4), H4(1), H5(3), H6(1), H7(1)	5	0.4	0.00122
DHS	23.28	112.88	5	H1(5)	1	0	0
Nan	22.78	108.38	10	H1(4), H8(1)	2	0.8	0.00329
Ding	19.55	110.36	2	H1(2)	1	0	0
XTBG	21.68	101.42	5	H9(3), H10(1), H11(1)	3	0.7	0.0058
Site3	15.02	98.58	4	H14(3), H15(1)	2	0.5	0.00763
Site10	13.47	102.18	5	H12(4), H13(1)	2	0.4	0.00061
TN	7.92	100.28	4	H14(4)	1	0	0
YN	−6.37	106.82	5	H16(1), H17(3), H18(1)	3	0.7	0.00336

Abbreviations: H, name of haplotypes with number of individuals; *h*, number of haplotypes; *Hd*, haplotype diversity; *N*, Number of sequences; *Pi*, nucleotide diversity.

Genomic DNA was extracted using The EasyPure Genomic DNA Extraction Kit (TransGen) after morphological observation and taking pictures under Leica EZ4 E stereo microscopes. Sequence data were obtained from *COI* mitochondrial genes (COI‐Jerrys: CAACATTTATTTTGATTTTTTGG and COI‐Pat: TCCAATGCACATATCTGCCATATTA) and the D2 domain of the 28S rRNA genes (28S‐F: ACCCGCTGAATTTAAGCATAT 28S‐R: TAGTTCACCATCTTTCGGGTC), using standard methods. These gene regions have been frequently used in phylogenetic studies of closely related species of fig wasps (Cruaud et al., 2012; Molbo et al., 2003; Yang et al., 2015). PCR amplification of COI and 28S was carried out in a 25 μL reaction volume using Premix primer 12.5 μL, ddH_2_O 7.5 μL, each primer 0.5 μL, and DNA 4 μL. The reaction of COI was optimized and programmed on an MJ Thermal Cycler (PTC 200) as one cycle of denaturation at 95°C for 5 min, 35 cycles of 30 s denaturation at 94°C, 60 s at a 50–54°C annealing temperature, and 45 s extension at 72°C, followed by 8 min extension at 72°C. And the reaction of the 28S as one cycle of denaturation at 95°C for 3 min, 34 cycles of 60 s denaturation at 94°C, 50 s at a 60°C annealing temperature, and 70 s extension at 72°C, followed by 7 min extension at 72°C. All amplified PCR products were purified using QIAquick spin columns (Qiagen) and were sequenced in an ABI 3730xl capillary sequencer using BIGDYE TERMINATOR V 3.1 chemistry (Applied Biosystems).

We aligned and edited our sequences using BioEdit version 7.0. 0 (Hall, 2004) and MEGA X (Kumar et al., 2018). We also checked for indications of pseudogenes such as multiple peaks in chromatograms, stop codons, or frameshift mutations according to the procedure suggested by Song et al. (2008). No evidence of pseudogenes or heteroplasmy was noted for mitochondrial regions. Phylogenetic analyses were performed on the datasets using maximum likelihood methods. Sequences of pollinating wasp associated with *F. hispida* in GenBank were also downloaded in this study. Maximum likelihood trees were constructed using MEGA X for COI and 28S both separately and combined and node supports were assessed based on 1000 bootstrap replicates. GenBank Accession numbers for all downloaded sequences were as Table S1. The model for the two genes and their combination was chosen using jModelTest2 (Darriba et al., 2012). HKY + G model was chosen for COI, the T92 model was chosen for 28S, and GTR + G model was chosen for the combined sequences. We used two *Ceratosolen notus* sequences (COI and 28S) as outgroups.

We used genetic distance (barcoding‐type) and morphological differences to delimit species. We calculated the Kimura‐2‐parameter (K2P) pairwise distance in MEGA X (Kumar et al., 2018) and plotted them in order to determine visually whether a barcode gap was present. To explore the number of operational taxonomic units (OTUs) that may represent potential cryptic species within *Ceratosolen* species, we employed the distance‐based barcode‐gap approach using the Automatic Barcode Gap Discovery (ABGD; Puillandre, Lambert, et al., 2012; Puillandre, Modica, et al., 2012). The ABGD method is based upon pairwise distance measures, accessible at https://bioinfo.mnhn.fr/abi/public/abgd/. With this method, the sequences are partitioned into groups or OTUs. The distance between two sequences from two different groups will always be larger than a given threshold distance (i.e., barcode gap). We used primary partitions as a principal for group definition. According to Puillandre, Lambert, et al. (2012) and Puillandre, Modica, et al. (2012), they are typically stable over a wider range of initial values, minimize the number of false positives (over split species), and are usually close to the number of groups described by taxonomists. Default values were used with the default settings (*P*_min_ = 0.001, *P*_max_ = 0.1, Steps = 10, X (relative gap width) = 1.5, Nb bins (for distance distribution = 20)) and Kimura 2‐parameter distances calculated in mega X. Pairwise sequence divergences were not calculated for 28S rRNA because no sequence variation was observed within subclades.

To demonstrate that we detected distinct species, multifocal color images were made using a Nikon SMZ25 microscope with a Nikon DSRi 2 digital camera system, and images were taken with a digital camera connected to a stereomicroscope (LIECA M205 FA, Leica Microsystem GmbH). Moreover, morphology of fig wasp in this study was compared with subspecies descriptions by Wiebes (1963). Polymorphism indices of the mtCOI sequences were calculated using the software DnaSP 5.0 (Librado & Rozas, 2009). Polymorphism indices included the number of haplotypes (h), haplotype diversity (Hd), and nucleotide diversity (Pi). For comparison, relationships among haplotypes were also estimated using a statistical parsimony network approach and created haplotype networks using R and “pegas” adapted from https://johnbhorne.wordpress.com/2016/09/15/still‐making‐haplotype‐networks‐the‐old‐way‐how‐to‐do‐it‐in‐r/. Finally, we plotted the geographic distributions of the pollinator species across 10 regional populations in R (Team, 2013).

## 3 RESULTS

**FIGURE 2:**
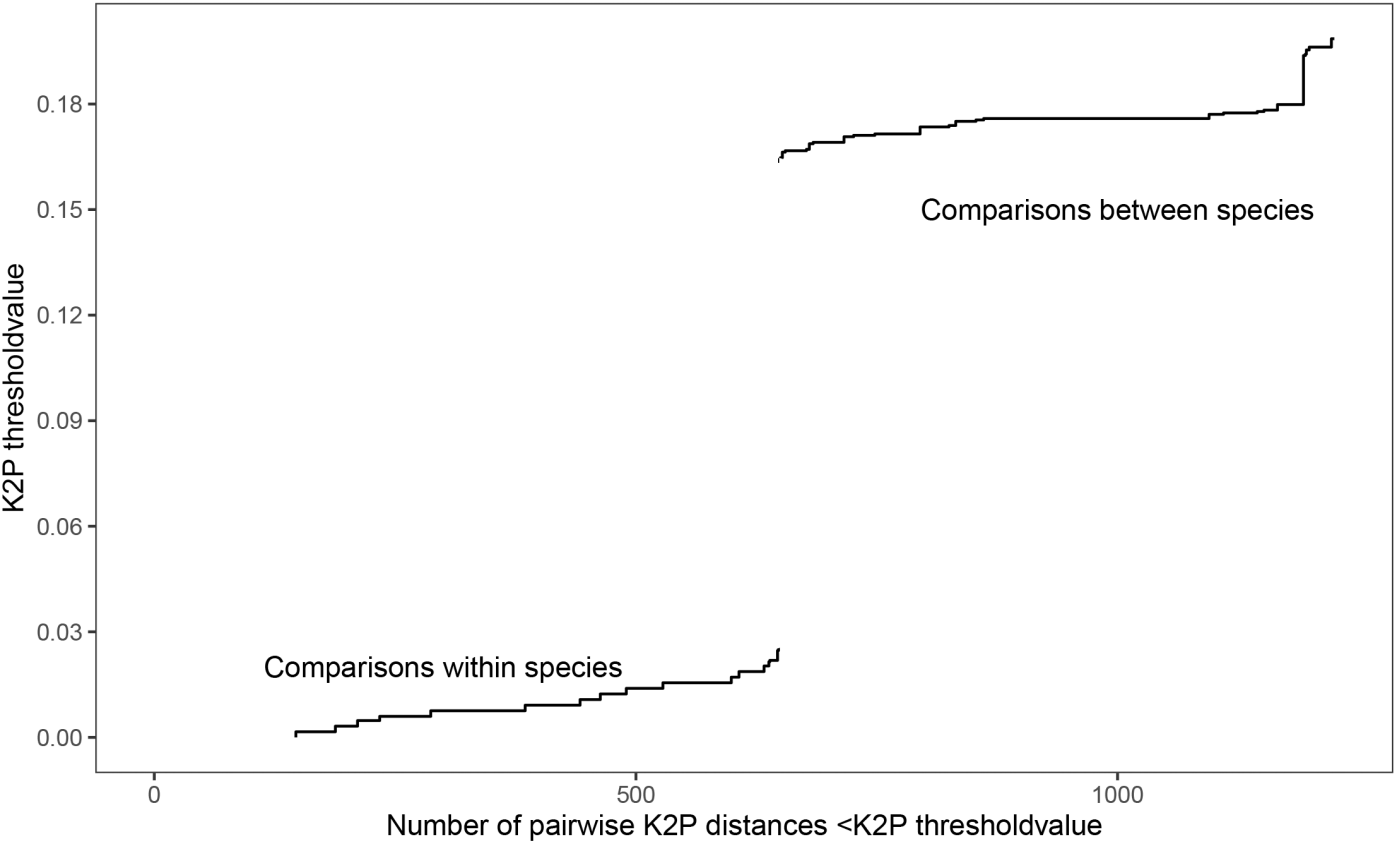
Cumulative distribution of Kimura pairwise genetic distances (K2P) for COI for *Ceratosolen* species associated with *Ficus hispida*.

We analyzed 50 sequences of *Ceratosolen* species associated with *F. hispida* from China and Southeast Asia (Table 1). The cumulative distribution of K2P distances for COI presents a significant barcoding gap between clades. K2P pairwise distances ranged from 0% to 2.83% within species and 16.449%–19.856% between different species (Figure 2). The ABGD method obtained four thresholds at 0.1%, 0.16%, 0.27%, and 0.46%, corresponding to 17, 10, 6, and 2 hypothetical species, respectively (Figure S1).

**FIGURE 3:**
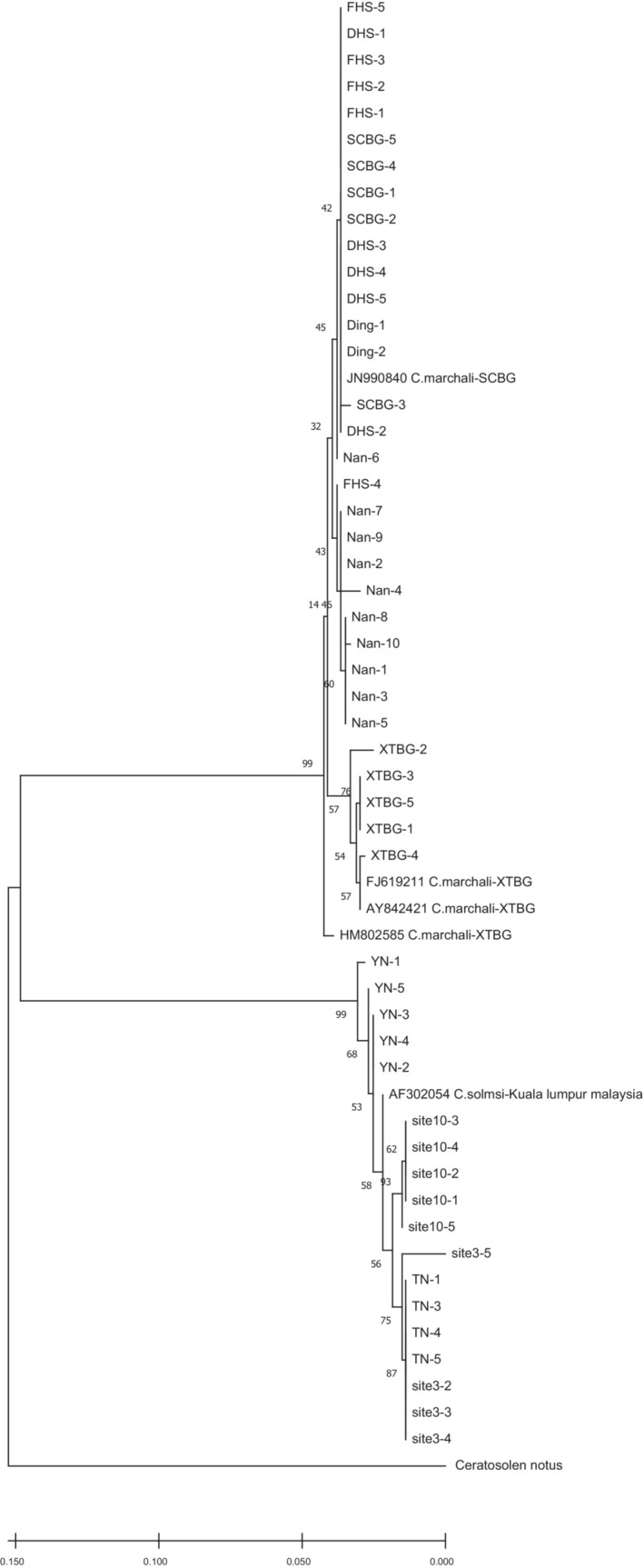
Maximum likelihood phylogenetic tree based on COI sequences of the *Ceratosolen* fig wasps associated with *Ficus hispida*, including the sequences in the study and sequences retrieved from NCBI.

**FIGURE 4:**
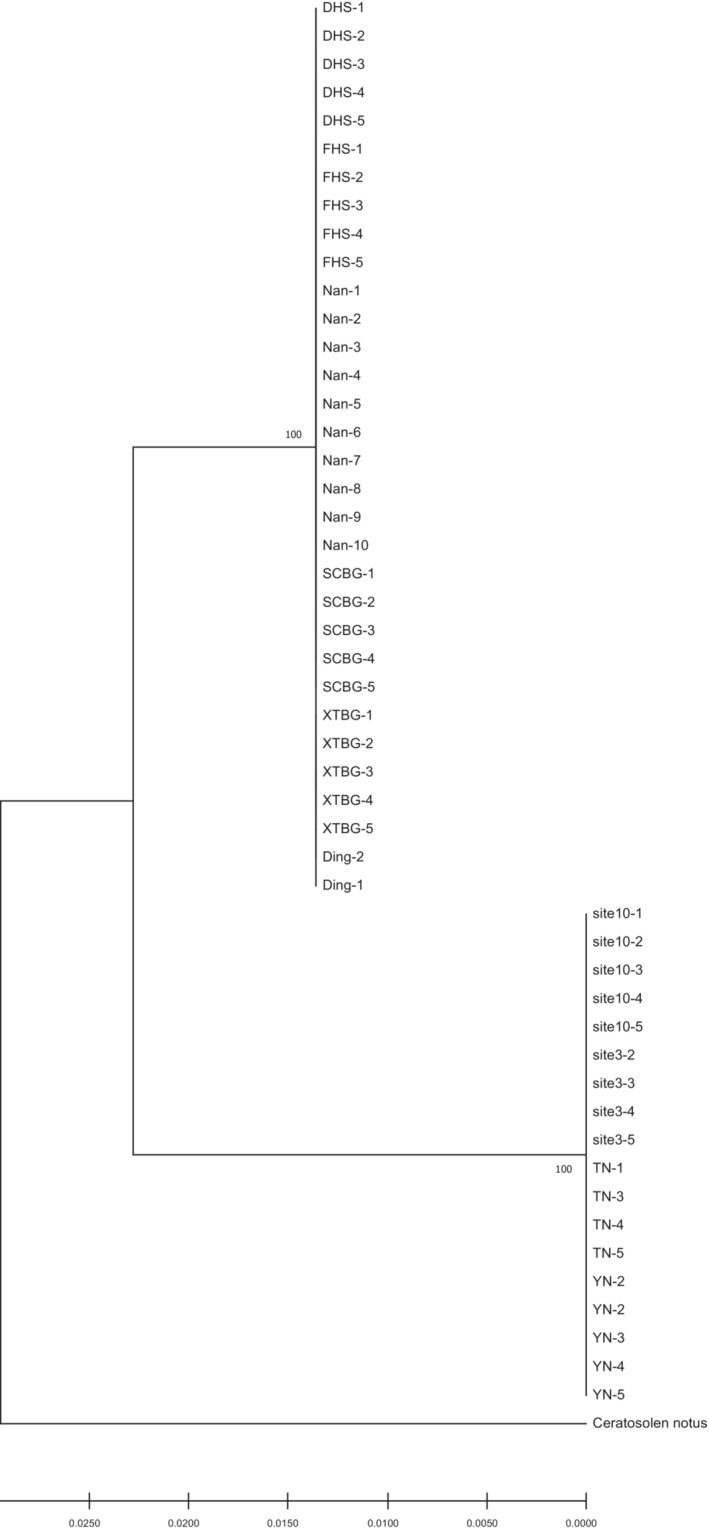
Maximum likelihood phylogenetic tree based on 28S sequences of the *Ceratosolen* fig wasps associated with *Ficus hispida*.

However, in contrast to the COI results (Figure 3), there is a marked lack of subclade structure in 28S sequences (Figure 4). In fact, it is so obvious that most individuals within each species have identical sequences, and there are substantial differences between species, making species placement unequivocal using this marker. Likewise, all species placements are congruent with those determined by COI analyses and combined sequences analyses, suggesting two species, *C. marchali* and *C. solmsi* (Figures 3 and 4, and Figure S2).

**FIGURE 5:**
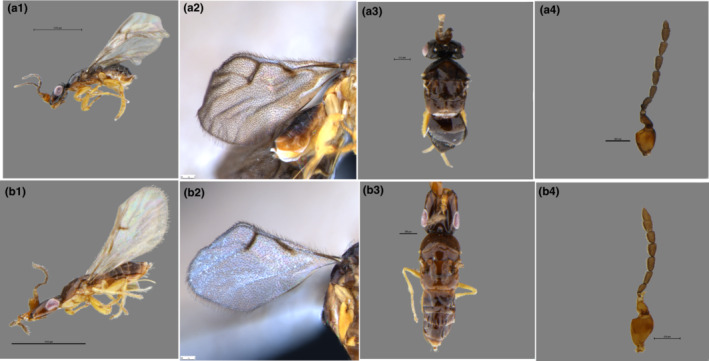
*C. marchali* (a1–a4) and *C. solmsi* (b1–b4). (a1, b1) Habitus, lateral view; (a2, b2) fore wing; (a3, b3) propodeum, dorsal view; (a4, b4) antenna, lateral view.

**FIGURE 6:**
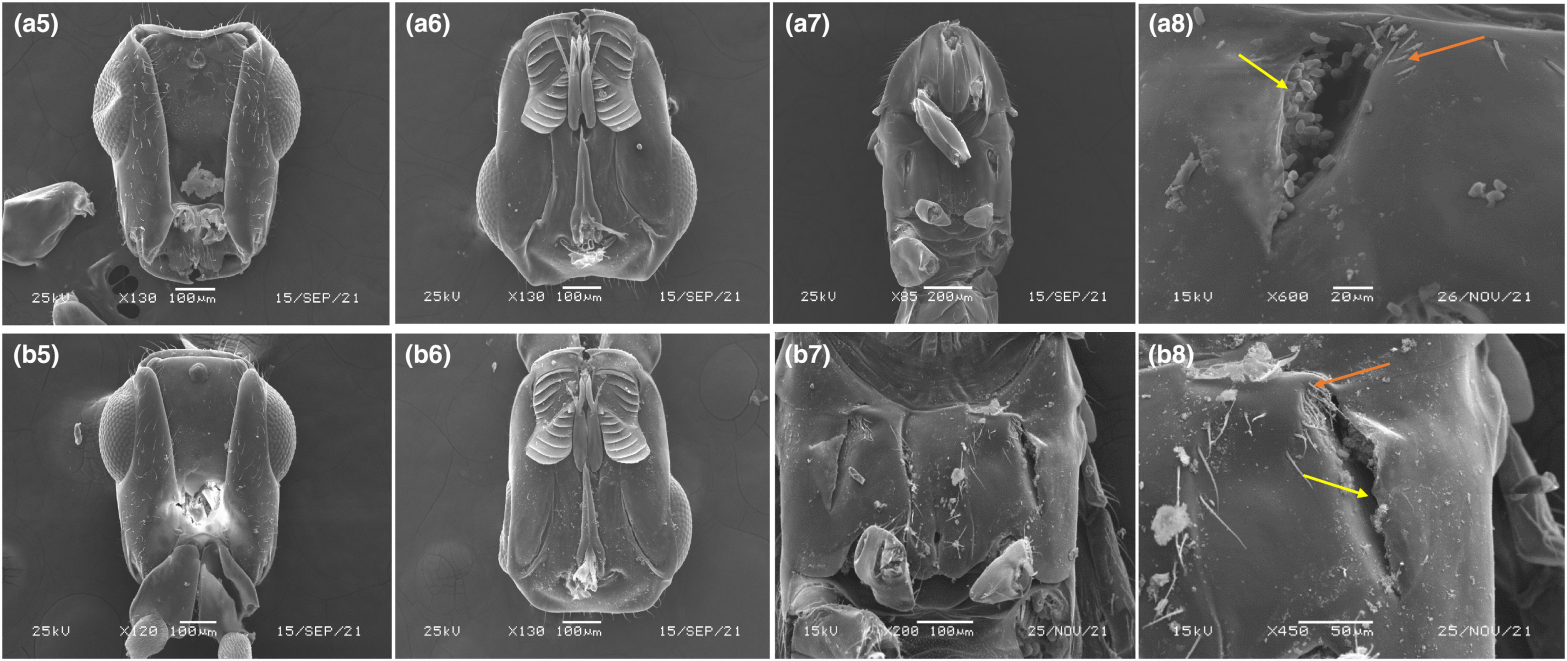
*C. marchali* (a5–a8) and *C. solmsi* (b5–b8). (a5, b5) Head, dorsal view; (a6, b6) Head, ventral view; (a7, b7) Mesosoma, ventral view; (a8, b8) pollen pocket, dorsal view.

The results of morphological identification showed that although the two wasp species are morphologically similar, the females of *C. marchali* and *C. solmsi* mainly differ in the following characters. The body color is yellow in both species, but it is darker in *C. marchali* (Figure 5a3,b3). As described by Wiebes (1963), the fore wings of *C. solmsi* are hyaline, while those of *C. marchali* are darker, with striae radiating from the stigma vein (Figure 5a2,b2). Besides, the two species differ in the morphological traits of female antennae and pollen pocket, two features that are directly related to the adaptation of pollinators to host figs (Ramírez Benavides, 1991, 1978). Fig wasp' antennae are responsible for perception of scent signals (Hossaert‐McKey et al., 2010). However, in *C. solmsi*, the 11th antennal segment is longer than the 10th, while in *C. marchali* it is shorter (Figure 5a4,b4). Although Wiebes (1963) also found differences in their antennae, he treated them as subspecies, probably mainly because he found intermediates between the different populations and lacked molecular evidence. Pollen pocket is a very important morphological characteristics to distinguish different wasp species (Deng et al., 2023; Weiblen, 2002). In this study, the pollen pocket of *C. marchali* is relatively shorter and wider, whereas those of *C. solmsi* is slender and seem to carry less pollen (Figure 6a8,b8). Therefore, combining morphological and molecular evidence, we can confirm that we detect two distinct species, rather than two subspecies.

Finally, the geographical distributions of the different haplotypes and associated diversity parameters of COI are given in Table 1, and the haplotype networks were given in Figure S3. From 50 mtDNA COI sequences (658 bp) from 10 sites, 18 different haplotypes were detected. Network profile of these mtDNA haplotypes clustered into two clades (Figure S3) and the average numbers of haplotypes were 2.2 (1–5) (Table 1). Table 2 and Figure S4 showed the distribution of pollinators belonging to each of the two *Ceratosolen* species associated with *F. hispida* at sites across our study locations.

**TABLE 2:** The species groups separated from the selected partition.

Location	Latitude	Longitude	Species
FHS	23.37	114.37	*C. marchali*
SCBG	23.28	113.58	*C. marchali*
DHS	23.28	112.88	*C. marchali*
Nan	22.78	108.38	*C. marchali*
Ding	19.55	110.36	*C. marchali*
XTBG	21.68	101.42	*C. marchali*
Site3	15.02	98.58	*C. solmsi*
Site10	13.47	102.18	*C. solmsi*
TN	7.92	100.28	*C. solmsi*
YN	−6.37	106.82	*C. solmsi*

## 4 DISCUSSION

This study focused on the genetic differentiation of *Ceratosolen* species associated with *F. hispida* in China and Southeast Asia. According to our species delimitation analyses, the genetic distance within the two *Ceratosolen* species ranged from 0% to 2.83% and between species from 16.449% to 19.856% (Figure 2). A study by Moe and Weiblen (2010) found that in *Ceratosolen*, genetic differences between sister species were large but similar to the Hymenoptera data, ranging from 7.6% to 20.4%, with a mean of 11.3 (±4%). Deep divergence was also identified in several pollinating wasp sequences of *F. hispida* from China and Southeast Asia. However, for the purpose of discussion, they referred to genetically divergent lineages as “cryptic species” (Moe & Weiblen, 2010). In this study, our results of morphological identification showed that the two wasp species differ in wing venation, body coloration, antennae shape, and pollen pocket (Figures 5 and 6). Both morphological and molecular evidence indicated that there are two *Ceratosolen* species within the study range, but not two subspecies associated with *F. hispida*.

Morphological and molecular results suggested that that pollinating wasp of *F. hispida* has diversified into two distinct species from southern China to southern Thailand to Indonesia (Figures 3, 5 and 6). Our sampling results showed that in southern and southwestern China, *F. hispida* is pollinated by *C. marchali*, whereas in southern Thailand and Indonesia, it is pollinated by *C. solmsi*, while for monoecious *F. racemosa*, it is pollinated by a single agaonid wasp species in China and continental South‐East Asia (Bain et al., 2016; Kobmoo et al., 2010). In contrast, for the short‐distance dispersed *F. hirta*, it is pollinated by nine parapatric pollinators in its continental Asian distribution (Yu et al., 2019). Indeed, this is the highest number of pollinators reported to date for any *Ficus* species. Therefore, our findings were generally in concordance with our expectations that among broadly sympatric Asian *Ficus* species, the number of pollinating fig wasp species with intermediately dispersed *F. hispida* is greater than that of long‐distance dispersed *F. racemosa* and smaller than that of locally dispersed *F. hirta* on the mainland.

Long‐distance wasp dispersal may prevent genetic and ecological differentiation within species, limiting opportunities for separation into differentiated populations and incipient species. These fig wasps that pollinate monoecious figs can drift in the wind for at least 30 km from neighboring areas (Harrison, 2003; Nason et al., 1998). A previous study confirmed that *Ceratosolen arabicus*, the pollinator of the monoecious African fig tree‐*Ficus sycomorus*, mediates directional pollen transfer between desert fig trees 160 km apart (Ahmed et al., 2009), and it also belongs to the group of *Ceratosolen* species that pollinate subsection *Sycomorus* (Kerdelhue et al., 1999). In the monoecious *F. racemosa*, no spatial genetic structure was found for fig wasps pollinating in *F. racemosa* (Moraceae) over a 1600 km stretch, and a limited structure of wasp genetic diversity was detected (Bain et al., 2016; Kobmoo et al., 2010). Hence, we speculate that the lack of local genetic structuring is an available feature in fig wasps pollinating monoecious *Ficus* species. The opposite may be true for the dioecious *Ficus* species presenting less gene flow. Firstly, in *F. hirta*, across its range, it displays clinal genetic variation and is pollinated by multiple parapatric species of *Valisia* (Yu et al., 2019). It is the first demonstration of the occurrence of parapatric pollinator species on a fig host displaying continuous genetic structure. *Ficus hirta*, an understory, dioecious shrub or tree (1.5–2.5 m), widely sympatric with *F. hispida*, is pollinated by Sp1 in eastern China and Sp2 in Yunnan (Yu et al., 2019). Secondly, in the creeping evergreen shrub *F. pumila*, the genetic structure of the pollinating wasp displays allopatric mainly distribution of the three species in Southeastern China (Chen et al., 2012). Thirdly, in the small free‐standing *F. septic*, the two polymorphic pollinating wasp species are structured into three vicariant black‐colored species and a fourth yellow‐colored species throughout the Philippines and Taiwan (Rodriguez et al., 2017), suggesting restricted gene flow between islands. In our study, both morphological and molecular results indicate that *F. hispida* is pollinated by two fig wasp species at our study sites (Figures 3, 4, 5, 6). All these studies suggested that the fig wasps from monoecious *Ficus* are more dispersed than that from dioecious *Ficus* (Nazareno et al., 2013). Dispersal of fig wasps is wind‐assisted (Harrison, 2003), whereas dioecious fig pollinators were more limited dispersal probably owing to reduced wind speeds.

In the fig‐fig wasp system, plant‐pollinator encounters are mediated by the cues of receptive odors (Proffit et al., 2020a; Soler et al., 2012; Zhang et al., 2020). While geographic differences in floral scent may be influenced by environmental factors (Farré‐Armengol et al., 2020), these differences may be driven by the genetics of the host plant, rather than by insect genetic structure (Deng et al., 2022; Deng, Buatois, et al., 2021), suggesting that plants evolve first and then the wasps have to adapt to local odors, consistent with our expectations of increased pollinator diversity increased with reduced gene flow of host fig species.

## AUTHOR CONTRIBUTIONS

**Xiaoxia Deng:** Methodology (equal); software (equal); writing – original draft (equal); writing – review and editing (equal). **Yaolin Liao:** Data curation (equal); formal analysis (equal). **Da‐Mien Wong:** Data curation (supporting); supervision (equal). **Hui Yu:** Conceptualization (equal); supervision (equal); writing – review and editing (equal).

## FUNDING INFORMATION

This work was supported by National Key R & D Program of China (2023YFE0107400), Science and Technology Projects in Guangzhou, the China Scholarship Council No. (2017)3109 and the National Natural Science Foundation of China (Grant No. 31971568).

## CONFLICT OF INTEREST STATEMENT

None declared.

## Supporting information


Figure S1.

Figure S2.

Figure S3.

Figure S4.

Table S1.

## Data Availability

COI sequences: https://figshare.com. Resource/doi: https://doi.org/10.6084/m9.figshare.19927880. 28S sequences: https://figshare.com. Resource/doi: https://doi.org/10.6084/m9.figshare.19926125.

## References

[ece310518-bib-0001] Ahmed, S. , Compton, S. G. , Butlin, R. K. , & Gilmartin, P. M. (2009). Wind‐borne insects mediate directional pollen transfer between desert fig trees 160 kilometers apart. Proceedings of the National Academy of Sciences of the United States of America, 106(48), 20342–20347. 19910534 10.1073/pnas.0902213106 PMC2787140

[ece310518-bib-0002] Alamo‐Herrera, C. R. , Arteaga, M. C. , Bello‐Bedoy, R. , & Rosas‐Pacheco, F. (2022). Pollen dispersal and genetic diversity of *Yucca valida* (Asparagaceae), a plant involved in an obligate pollination mutualism. Biological Journal of the Linnean Society, 136(2), 364–374.

[ece310518-bib-0003] Bain, A. , Borges, R. M. , Chevallier, M. H. , Vignes, H. , Kobmoo, N. , Peng, Y. Q. , Cruaud, A. , Rasplus, J. Y. , Kjellberg, F. , & Hossaert‐Mckey, M. (2016). Geographic structuring into vicariant species‐pairs in a wide‐ranging, high‐dispersal plant–insect mutualism: The case of *Ficus racemosa* and its pollinating wasps. Evolutionary Ecology, 30(4), 663–684.

[ece310518-bib-0006] Barrett, S. C. (2003). Mating strategies in flowering plants: The outcrossing‐selfing paradigm and beyond. Philosophical Transactions of the Royal Society B, 358(1434), 991–1004. 10.1098/rstb.2003.1301 PMC1693196 12831464

[ece310518-bib-0007] Bascompte, J. , Jordano, P. , & Olesen, J. M. (2006). Asymmetric coevolutionary networks facilitate biodiversity maintenance. Science, 312(5772), 431–433. 16627742 10.1126/science.1123412

[ece310518-bib-0008] Benton, M. J. , Wilf, P. , & Sauquet, H. (2022). The angiosperm terrestrial revolution and the origins of modern biodiversity. New Phytologist, 233(5), 2017–2035. 34699613 10.1111/nph.17822

[ece310518-bib-0009] Berg, C. C. (1989). Classification and distribution of *Ficus* . Experientia, 45(7), 605–611.

[ece310518-bib-0010] Berg, C. C. , & Corner, E. J. H. (2005). Moraceae: Ficeae. Flora Malesiana Series I, 17(2), 1–70.

[ece310518-bib-0011] Borges, R. M. (2016). On the air: Broadcasting and reception of volatile messages in brood‐site pollination mutualisms. In J. D Blonde & R. Glinwood (Eds.), Deciphering chemical language of plant communication (pp. 227–255). Springer.

[ece310518-bib-0013] Carvalho, M. R. , Jaramillo, C. , de la Parra, F. , Caballero‐Rodríguez, D. , Herrera, F. , Wing, S. , & Silvestro, D. (2021). Extinction at the end‐cretaceous and the origin of modern neotropical rainforests. Science, 372(6537), 63–68. 33795451 10.1126/science.abf1969

[ece310518-bib-0014] Chen, Y. , Compton, S. G. , Liu, M. , & Chen, X. Y. (2012). Fig trees at the northern limit of their range: The distributions of cryptic pollinators indicate multiple glacial refugia. Molecular Ecology, 21(7), 1687–1701. 22335780 10.1111/j.1365-294X.2012.05491.x

[ece310518-bib-0015] Chen, Y. , Jiang, Z.‐X. , Compton, S. G. , Liu, M. , & Chen, X.‐Y. (2011). Genetic diversity and differentiation of the extremely dwarf *Ficus tikoua* in southwestern China. Biochemical Systematics and Ecology, 39(4–6), 441–448.

[ece310518-bib-0017] Corlett, R. T. (2006). Figs (*Ficus*, Moraceae) in urban Hong Kong, South China 1. Biotropica: The Journal of Biology and Conservation, 38(1), 116–121.

[ece310518-bib-0018] Cornille, A. , Underhill, J. G. , Cruaud, A. , Hossaert‐McKey, M. , Johnson, S. D. , Tolley, K. A. , Kjellberg, F. , van Noort, S. , & Proffit, M. (2012). Floral volatiles, pollinator sharing and diversification in the fig‐wasp mutualism: Insights from *Ficus natalensis*, and its two wasp pollinators (South Africa). Proceedings of the Royal Society B: Biological Sciences, 279(1734), 1731–1739. 10.1098/rspb.2011.1972 PMC3297447 22130605

[ece310518-bib-0019] Cousins, S. R. , & Witkowski, E. T. F. (2017). African cycad ecology, ethnobotany and conservation: A synthesis. The Botanical Review, 83(2), 152–194.

[ece310518-bib-0020] Cresswell, J. E. , Osborne, J. L. , & Bell, S. A. (2002). A model of pollinator‐mediated gene flow between plant populations with numerical solutions for bumblebees pollinating oilseed rape. Oikos, 98(3), 375–384.

[ece310518-bib-0021] Cruaud, A. , Rønsted, N. , Chantarasuwan, B. , Chou, L. S. , Clement, W. L. , Couloux, A. , Cousins, B. , Genson, G. , Harrison, R. D. , Hanson, P. E. , Hossaert‐McKey, M. , Jabbour‐Zahab, R. , Jousselin, E. , Kerdelhué, C. , Kjellberg, F. , Lopez‐Vaamonde, C. , Peebles, J. , Peng, Y.‐Q. , Pereira, R. A. S. , … Savolainen, V. (2012). An extreme case of plant–insect codiversification: Figs and fig‐pollinating wasps. Systematic Biology, 61(6), 1029–1047. 22848088 10.1093/sysbio/sys068 PMC3478567

[ece310518-bib-0022] Darriba, D. , Taboada, G. L. , Doallo, R. , & Posada, D. (2012). jModelTest 2: More models, new heuristics and parallel computing. Nature Methods, 9(8), 772. 10.1038/nmeth.2109 PMC4594756 22847109

[ece310518-bib-0024] Deng, X. , Buatois, B. , Peng, Y.‐Q. , Yu, H. , Cheng, Y. , Kjellberg, F. , & Proffit, M. (2021). Plants are the drivers of geographic variation of floral scents in a highly specialized pollination mutualism: a study of Ficus hirta in China . Res Square.(priprint).

[ece310518-bib-0025] Deng, X. , Chen, L. , Tian, E. , Zhang, D. , Wattan, T. , Yu, H. , Kjellberg, F. , & Segar, S. T. (2021). Low host specificity and broad geographic ranges in a community of parasitic non‐pollinating fig wasps (Sycoryctinae; Chalcidoidea). Journal of Animal Ecology, 90(7), 1678–1690. 33738802 10.1111/1365-2656.13483

[ece310518-bib-0026] Deng, X. , Cheng, Y. , Peng, Y. Q. , Yu, H. , Proffit, M. , & Kjellberg, F. (2022). Overlaps in olfactive signalling coupled with geographic variation may result in localised pollinator sharing between closely related *Ficus* species. BMC Ecology and Evolution, 22(1), 97. 35964015 10.1186/s12862-022-02055-0 PMC9375327

[ece310518-bib-0027] Deng, X. , Liao, Y. , Liu, W. , & Yu, H. (2023). The coexistence of two related fig wasp species sharing the same host fig species across a broad geographical area. Acta Oecologica, 118, 103885.

[ece310518-bib-0028] Dufaÿ, M. , Hossaert‐McKey, M. , & Anstett, M. C. (2003). When leaves act like flowers: How dwarf palms attract their pollinators. Ecology Letters, 6(1), 28–34.

[ece310518-bib-0029] Ennos, R. (1994). Estimating the relative rates of pollen and seed migration among plant populations. Heredity, 72(3), 250–259.

[ece310518-bib-0030] Farré‐Armengol, G. , Fernandez‐Martinez, M. , Filella, I. , Junker, R. R. , & Penuelas, J. (2020). Deciphering the biotic and climatic factors that influence floral scents: A systematic review of floral volatile emissions. Frontiers in Plant Science, 11, 1154. 32849712 10.3389/fpls.2020.01154 PMC7412988

[ece310518-bib-0031] Grison‐Pigé, L. , Bessière, J.‐M. , & Hossaert‐McKey, M. (2002). Specific attraction of fig‐pollinating wasps: Role of volatile compounds released by tropical figs. Journal of Chemical Ecology, 28(2), 283–295. 11925068 10.1023/a:1017930023741

[ece310518-bib-0033] Hall, T. A. (2004). BioEdit sequence alignment editor 7.0. 1. Isis Pharmaceuticals.

[ece310518-bib-0034] Harrison, R. D. (2003). Fig wasp dispersal and the stability of a keystone plant resource in Borneo. Proceedings of the Royal Society B: Biological Sciences, 270(suppl_1), S76–S79. 10.1098/rsbl.2003.0018 PMC1698007 12952642

[ece310518-bib-0035] Harrison, R. D. (2005). Figs and the diversity of tropical rainforests. Bioscience, 55(12), 1053–1064.

[ece310518-bib-0036] Harrison, R. D. , & Rasplus, J. Y. (2006). Dispersal of fig pollinators in Asian tropical rain forests. Journal of Tropical Ecology, 22(6), 631–639.

[ece310518-bib-0037] Harrison, R. D. , Ronsted, N. , & Peng, Y. Q. (2008). Foreword fig and fig wasp biology: A perspective from the east. Symbiosis, 45, 1–8.

[ece310518-bib-0038] Harrison, R. D. , & Yamamura, N. (2003). A few more hypotheses for the evolution of dioecy in figs (*Ficus*, Moraceae). Oikos, 100(3), 628–635.

[ece310518-bib-0040] Herre, E. A. , Jander, K. C. , & Machado, C. A. (2008). Evolutionary ecology of figs and their associates: Recent progress and outstanding puzzles. Annual Review of Ecology, Evolution, and Systematics, 39, 439–458.

[ece310518-bib-0041] Hossaert‐Mckey, M. , Gibernau, M. , & Frey, J. E. (1994). Chemosensory attraction of fig wasps to substances produced by receptive figs. Entomologia Experimentalis et Applicata, 70(2), 185–191.

[ece310518-bib-0042] Hossaert‐McKey, M. , Soler, C. , Schatz, B. , & Proffit, M. (2010). Floral scents: Their roles in nursery pollination mutualisms. Chemoecology, 20(2), 75–88.

[ece310518-bib-0043] Huang, J. F. , Li, S. Q. , Xu, R. , & Peng, Y. Q. (2023). East–west genetic differentiation across the Indo‐Burma hotspot: Evidence from two closely related dioecious figs. BMC Plant Biology, 23(1), 321. 37322436 10.1186/s12870-023-04324-6 PMC10273766

[ece310518-bib-0044] Jansen‐Gonzalez, S. , Teixeira, S. P. , & Pereira, R. A. S. (2012). Mutualism from the inside: Coordinated development of plant and insect in an active pollinating fig wasp. Arthropod‐Plant Interactions, 6, 601–609.

[ece310518-bib-0045] Kawakita, A. , & Kato, M. (2009). Repeated independent evolution of obligate pollination mutualism in the Phyllantheae‐*Epicephala* association. Proceedings of the Royal Society B: Biological Sciences, 276(1656), 417–426. 10.1098/rspb.2008.1226 PMC2664352 18948251

[ece310518-bib-0046] Kerdelhue, C. , Clainche, I. L. , & Rasplus, J. Y. (1999). Molecular phylogeny of the *Ceratosolen* species pollinating *Ficus* of the subgenus *Sycomorus sensu* stricto: Biogeographical history and origins of the species‐specificity breakdown cases. Molecular Phylogenetics and Evolution, 11(3), 401–414. 10196081 10.1006/mpev.1998.0590

[ece310518-bib-0047] Kobmoo, N. , Hossaert‐McKey, M. , Rasplus, J. Y. , & Kjellberg, F. (2010). *Ficus racemosa* is pollinated by a single population of a single agaonid wasp species in continental South‐East Asia. Molecular Ecology, 19(13), 2700–2712. 20561201 10.1111/j.1365-294X.2010.04654.x

[ece310518-bib-0048] Kuaraksa, C. , Elliott, S. , & Hossaert‐Mckey, M. (2012). The phenology of dioecious *Ficus* spp. tree species and its importance for forest restoration projects. Forest Ecology and Management, 265, 82–93.

[ece310518-bib-0049] Kumar, S. , Stecher, G. , Li, M. , Knyaz, C. , & Tamura, K. (2018). MEGA X: Molecular evolutionary genetics analysis across computing platforms. Molecular Biology and Evolution, 35(6), 1547–1549. 29722887 10.1093/molbev/msy096 PMC5967553

[ece310518-bib-0050] Lee, S. H. , Ng, A. B. C. , Ong, K. H. , O'Dempsey, T. , & Tan, H. T. W. (2013). The status and distribution of *Ficus hispida* Lf (Moraceae) in Singapore. Nature in Singapore, 6, 85–90.

[ece310518-bib-0051] Librado, P. , & Rozas, J. (2009). DnaSP v5: A software for comprehensive analysis of DNA polymorphism data. Bioinformatics, 25(11), 1451–1452. 19346325 10.1093/bioinformatics/btp187

[ece310518-bib-0053] Liu, M. , Compton, S. G. , Peng, F. E. , Zhang, J. , & Chen, X. Y. (2015). Movements of genes between populations: Are pollinators more effective at transferring their own or plant genetic markers? Proceedings of the Royal Society B: Biological Sciences, 282(1808), 20150290. 10.1098/rspb.2015.0290 PMC4455804 25948688

[ece310518-bib-0054] Liu, M. , Zhang, J. , Chen, Y. , Compton, S. G. , & Chen, X. Y. (2013). Contrasting genetic responses to population fragmentation in a coevolving fig and fig wasp across a mainland‐Island archipelago. Molecular Ecology, 22(17), 4384–4396. 23879300 10.1111/mec.12406

[ece310518-bib-0057] Moe, A. M. , & Weiblen, G. D. (2010). Molecular divergence in allopatric *Ceratosolen* (Agaonidae) pollinators of geographically widespread *Ficus* (Moraceae) species. Annals of the Entomological Society of America, 103(1), 1025–1037.

[ece310518-bib-0058] Molbo, D. , Machado, C. A. , Sevenster, J. G. , Keller, L. , & Herre, E. A. (2003). Cryptic species of fig‐pollinating wasps: Implications for the evolution of the fig–wasp mutualism, sex allocation, and precision of adaptation. Proceedings of the National Academy of Sciences of the United States of America, 100(10), 5867–5872. 12714682 10.1073/pnas.0930903100 PMC156293

[ece310518-bib-0059] Nason, J. D. , Herre, E. A. , & Hamrick, J. L. (1998). The breeding structure of a tropical keystone plant resource. Nature, 391(6668), 685–687.

[ece310518-bib-0060] Nazareno, A. G. , Alzate‐Marin, A. L. , & Pereira, R. A. (2013). Dioecy, more than monoecy, affects plant spatial genetic structure: The case study of *Ficus* . Ecology and Evolution, 3(10), 3495–3508. 24223285 10.1002/ece3.739 PMC3797494

[ece310518-bib-0061] Okamoto, T. , Kawakita, A. , Goto, R. , Svensson, G. P. , & Kato, M. (2013). Active pollination favours sexual dimorphism in floral scent. Proceedings of the Royal Society B: Biological Sciences, 280(1772), 20132280. 10.1098/rspb.2013.2280 PMC3813343 24266037

[ece310518-bib-0062] Proffit, M. , Chen, C. , Soler, C. , Bessière, J. M. , Schatz, B. , & Hossaert‐McKey, M. (2009). Can chemical signals, responsible for mutualistic partner encounter, promote the specific exploitation of nursery pollination mutualisms?–the case of figs and fig wasps. Entomologia Experimentalis et Applicata, 131(1), 46–57.

[ece310518-bib-0063] Proffit, M. , Lapeyre, B. , Buatois, B. , Deng, X. , Arnal, P. , Gouzerh, F. , Carrasco, D. , & Hossaert‐McKey, M. (2020). Chemical signal is in the blend: Bases of plant‐pollinator encounter in a highly specialized interaction. Scientific Reports, 10, 1–11. 32572098 10.1038/s41598-020-66655-w PMC7308319

[ece310518-bib-0064] Proffit, M. , Schatz, B. , Borges, R. M. , & Hossaert‐McKey, M. (2007). Chemical mediation and niche partitioning in non‐pollinating fig‐wasp communities. Journal of Animal Ecology, 76(2), 296–303. 17302837 10.1111/j.1365-2656.2007.01213.x

[ece310518-bib-0065] Puillandre, N. , Lambert, A. , Brouillet, S. , & Achaz, G. (2012). ABGD, automatic barcode gap discovery for primary species delimitation. Molecular Ecology, 21(8), 1864–1877. 21883587 10.1111/j.1365-294X.2011.05239.x

[ece310518-bib-0066] Puillandre, N. , Modica, M. V. , Zhang, Y. , Sirovich, L. , Boisselier, M. C. , Cruaud, C. , Holford, M. , & Samadi, S. (2012). Large‐scale species delimitation method for hyperdiverse groups. Molecular Ecology, 21(11), 2671–2691. 22494453 10.1111/j.1365-294X.2012.05559.x

[ece310518-bib-0067] R Core Team . (2013). R: A language and environment for statistical computing. R Foundation for Statistical Computing.

[ece310518-bib-0068] Ramírez Benavides, W. (1991). Evolution of the mandibular appendage in fig wasps (Hymenoptera: Agaonidae). Revista de Biologia Tropical., 39(1), 87–95.

[ece310518-bib-0069] Ramírez‐Benavides, W. (1978). Evolution of mechanisms to carry pollen in Agaonidae (Hymenoptera: Chalcidoidea). Tijdschrift voor Entomologie, 121(6), 279–293.

[ece310518-bib-0070] Rodriguez, L. J. , Bain, A. , Chou, L.‐S. , Conchou, L. , Cruaud, A. , Gonzales, R. , Hossaert‐McKey, M. , Rasplus, J.‐Y. , Tzeng, H.‐Y. , & Kjellberg, F. (2017). Diversification and spatial structuring in the mutualism between *Ficus septica* and its pollinating wasps in insular South East Asia. BMC Evolutionary Biology, 17(1), 1–12. 28851272 10.1186/s12862-017-1034-8 PMC5576367

[ece310518-bib-0071] Sakai, S. (2002). A review of brood‐site pollination mutualism: Plants providing breeding sites for their pollinators. Journal of Plant Research, 115(3), 161–168. 12579365 10.1007/s102650200021

[ece310518-bib-0072] Shi, T. , Toda, M. J. , Takano, K. T. , Yafuso, M. , Suwito, A. , Wong, S. Y. , Shang, S.‐Q. , & Gao, J.‐J. (2019). A review of taxonomy and flower‐breeding ecology of the *Colocasiomyia toshiokai* species group (Diptera: Drosophilidae), with description of a new species from Indonesia. European Journal of Entomology, 116, 341–361.

[ece310518-bib-0073] Soler, C. , Hossaert‐McKey, M. , Buatois, B. , Bessière, J. M. , Schatz, B. , & Proffit, M. (2011). Geographic variation of floral scent in a highly specialized pollination mutualism. Phytochemistry, 72(1), 74–81. 21109272 10.1016/j.phytochem.2010.10.012

[ece310518-bib-0074] Soler, C. C. , Proffit, M. , Bessière, J. M. , Hossaert‐McKey, M. , & Schatz, B. (2012). Evidence for intersexual chemical mimicry in a dioecious plant. Ecology Letters, 15(9), 978–985. 22762353 10.1111/j.1461-0248.2012.01818.x

[ece310518-bib-0075] Song, H. , Buhay, J. E. , Whiting, M. F. , & Crandall, K. A. (2008). Many species in one: DNA barcoding overestimates the number of species when nuclear mitochondrial pseudogenes are coamplified. Proceedings of the National Academy of Sciences of the United States of America, 105(36), 13486–13491. 18757756 10.1073/pnas.0803076105 PMC2527351

[ece310518-bib-0077] Suinyuy, T. N. , Donaldson, J. S. , & Johnson, S. D. (2015). Geographical matching of volatile signals and pollinator olfactory responses in a cycad brood‐site mutualism. Proceedings of the Royal Society B: Biological Sciences, 282(1816), 20152053. 10.1098/rspb.2015.2053 PMC4614789 26446814

[ece310518-bib-0078] Toon, A. , Terry, L. I. , Tang, W. , Walter, G. H. , & Cook, L. G. (2020). Insect pollination of cycads. Austral Ecology, 45(8), 1033–1058.

[ece310518-bib-0079] Verkerke, W. (1986). Anatomy of *Ficus ottoniifolia* (Moraceae) syconia and its role in the fig‐fig wasp symbiosis. Proceedings of the Koninklijke Nederlandse Akademie van Wetenschappen Series C Biological and Medical Sciences, 89, 443–470.

[ece310518-bib-0080] Verkerke, W. (1987). Synconial anatomy of *Ficus asperifolia* (Moraceae), a gynodioecious tropical fig. Proceedings of the Koninklijke Nederlandse Akademie van Wetenschappen Series C Biological and Medical Sciences, 90, 461–492.

[ece310518-bib-0082] Wang, R. , Yang, Y. , Jing, Y. , et al. (2021). Molecular mechanisms of mutualistic and antagonistic interactions in a plant–pollinator association. Nature Ecology & Evolution, 5, 1–13. 34002050 10.1038/s41559-021-01469-1

[ece310518-bib-0083] Ware, A. B. , & Compton, S. G. (1994). Dispersal of adult female fig wasps: 1. Arrivals and departures. Entomologia Experimentalis et Applicata, 73(3), 221–229.

[ece310518-bib-0084] Weiblen, G. D. (2002). How to be a fig wasp. Annual Review of Entomology, 47(1), 299–330. 10.1146/annurev.ento.47.091201.145213 11729077

[ece310518-bib-0092] Wiebes, J. T. (1963). Taxonomy and host preference of Indo‐Australian fig wasps of the genus Ceratosolen (Agaonidae). Tijdschrift voor Entomologie, 106, 1–112.

[ece310518-bib-0085] Willmott, K. R. , Willmott, J. C. R. , Elias, M. , & Jiggins, C. D. (2017). Maintaining mimicry diversity: Optimal warning colour patterns differ among microhabitats in Amazonian clearwing butterflies. Proceedings of the Royal Society B: Biological Sciences, 284(1855), 20170744. 10.1098/rspb.2017.0744 PMC5454276 28539522

[ece310518-bib-0086] Yang, D. R. , Peng, Y. , Song, Q. , & Zhang, G. (2002). Pollination biology of *Ficus hispida* in the tropical rainforests of Xishuangbanna, China. Acta Botanical Sinica, 44(5), 519–526.

[ece310518-bib-0087] Yang, L. Y. , Machado, C. A. , Dang, X. D. , Peng, Y. Q. , Yang, D. R. , Zhang, D. Y. , & Liao, W. J. (2015). The incidence and pattern of copollinator diversification in dioecious and monoecious figs. Evolution, 69(2), 294–304. 25495152 10.1111/evo.12584 PMC4328460

[ece310518-bib-0088] Yu, H. , Tian, E. , Zheng, L. , Deng, X. , Cheng, Y. , Chen, L. , Wu, W. , Tanming, W. , Zhang, D. , Compton, S. G. , & Kjellberg, F. (2019). Multiple parapatric pollinators have radiated across a continental fig tree displaying clinal genetic variation. Molecular Ecology, 28(9), 2391–2405. 30753744 10.1111/mec.15046

[ece310518-bib-0089] Zavodna, M. , Arens, P. , Van Dijk, P. J. , Partomihardjo, T. , Vosman, B. , & Van Damme, J. M. (2005). Pollinating fig wasps: Genetic consequences of Island recolonization. Journal of Evolutionary Biology, 18(5), 1234–1243. 16135119 10.1111/j.1420-9101.2005.00937.x

[ece310518-bib-0090] Zhang, X. , Wang, G. , Zhang, S. , Chen, S. , Wang, Y. , Wen, P. , Ma, X. , Shi, Y. , Qi, R. , Yang, Y. , Liao, Z. , Lin, J. , Lin, J. , Xu, X. , Chen, X. , Xu, X. , Deng, F. , Zhao, L. , Lee, Y. L. , … Ming, R. (2020). Genomes of the banyan tree and pollinator wasp provide insights into fig‐wasp coevolution. Cell, 183(4), 875–889. 33035453 10.1016/j.cell.2020.09.043

[ece310518-bib-0091] Zheng, L. (2015). *Phylogeography of Ficus hirta Vahl. in Southeast Asia* [Master's thesis of University of Chinese Academy of Sciences].

