# Supplementary figures and images for: The genetic structuring in pollinating wasps of Ficus hispida in continental Asia
Source: Ecol Evol. 2023 Sep 20;13(9):e10518. doi: 10.1002/ece3.10518 (PMC10511832; doi:10.1002/ece3.10518)

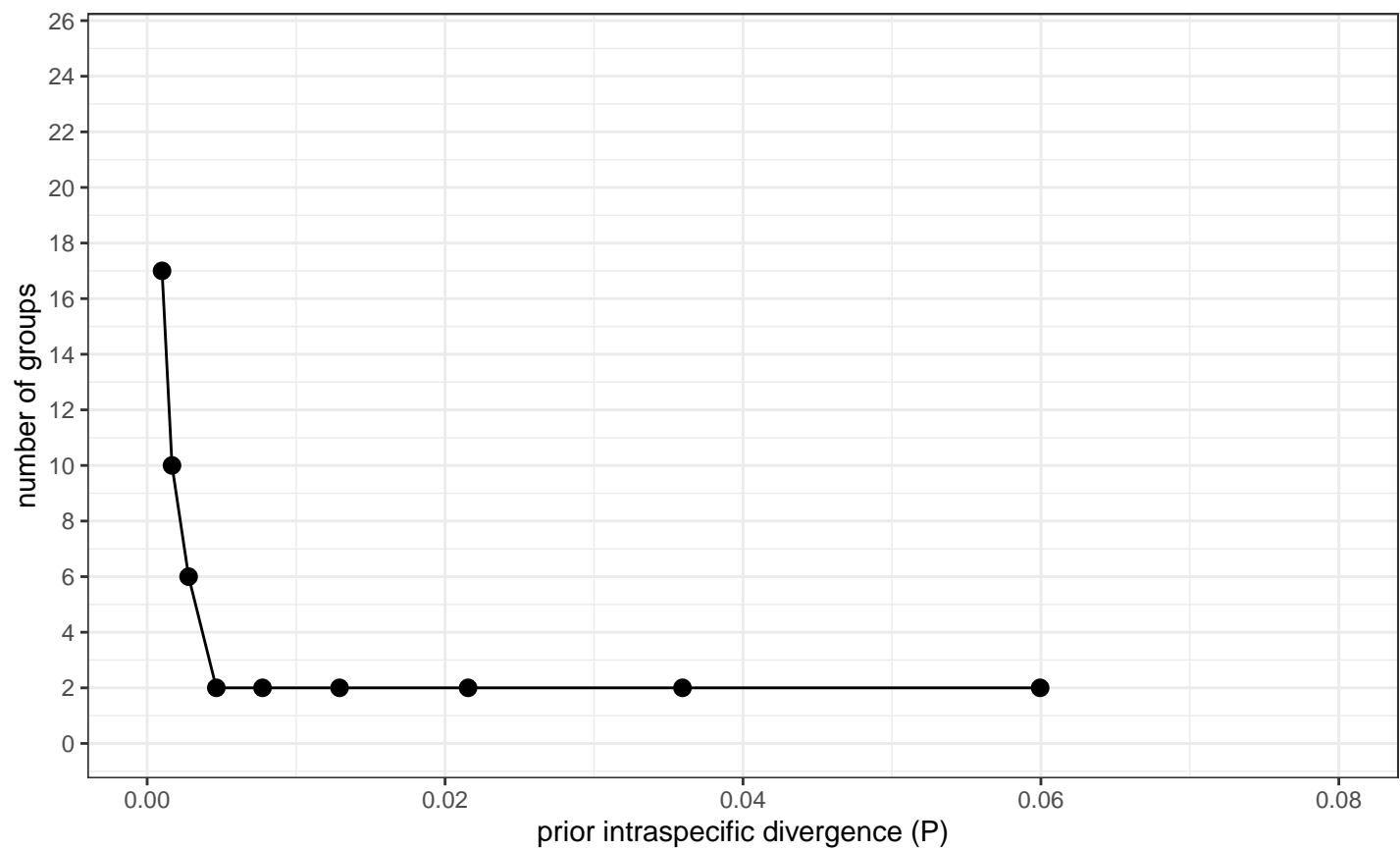

Supplement: Supplementary file 1 — Figure S1. [file ECE3-13-e10518-s003.pdf]

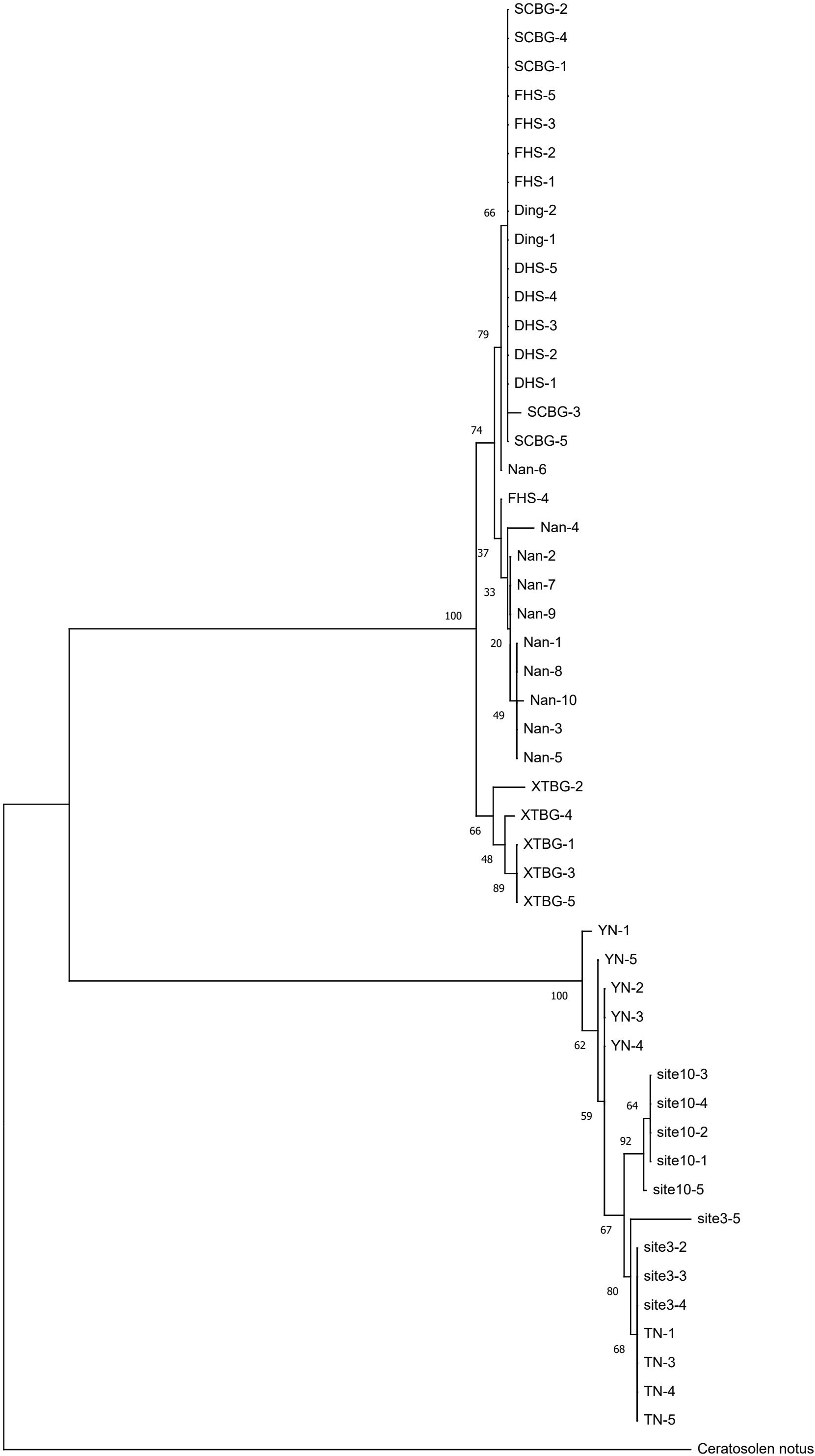

0.060

0.040

0.020

0.000

Supplement: Supplementary file 2 — Figure S2. [file ECE3-13-e10518-s002.pdf]

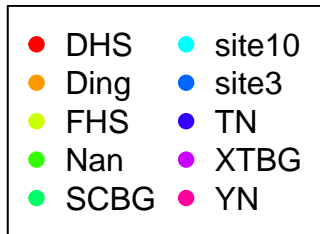

Supplement: Supplementary file 3 — Figure S3. [file ECE3-13-e10518-s005.pdf]

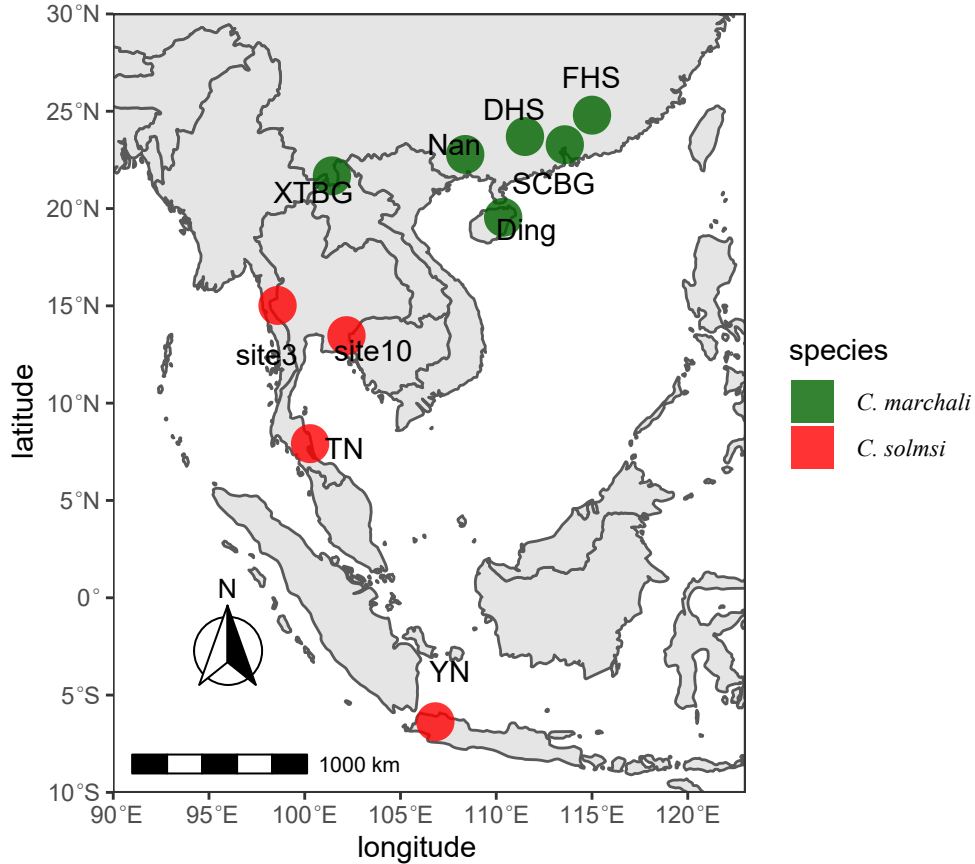

Supplement: Supplementary file 4 — Figure S4. [file ECE3-13-e10518-s001.pdf]
