# Supplementary material for: The genetic structuring in pollinating wasps of Ficus hispida in continental Asia
Source: Ecol Evol. 2023 Sep 20;13(9):e10518. doi: 10.1002/ece3.10518 (PMC10511832; doi:10.1002/ece3.10518)
Supplement: Supplementary file 5 — Table S1. [file ECE3-13-e10518-s004.docx]

Table S1 GenBank Acession numbers for all downloaded sequences of pollinating wasp associated with *F. hispida*.

| Country/region | GenBank Acession numbers of COI sequence | GenBank Acession numbers of 28S sequence |
| --- | --- | --- |
| XTBG, China | FJ619211 (Xuand Yang ,unpublished)  AY842421 (Jiang et al., 2006) HM802585 (Yang et al., 2015) | HM802741(Yang et al., 2015) |
| *Kuala lumpur,malaysia* | AF302054 (Murray, 1987) |  |
| SCBG, China | JN990840 (Tian and Yu, 2013) |  |

Jiang, Z.F., Huang, D.W., Zhu, C.D., Zhen, W.Q., 2006. New insights into the phylogeny of fig pollinators using Bayesian analyses. Molecular phylogenetics and evolution 38, 306-315.

Murray, M.G., 1987. The closed environment of the fig receptacle and its influence on male conflict in the Old World fig wasp, Philotrypesis pilosa. Animal Behaviour 35, 488-506.

Tian, E.-W., Yu, H., 2013. A Simple and Rapid Dna Extraction Protocol of Small Insects for Pcr Amplification. Entomological News 123, 303.

Xiao, J.H., Wang, N.X., Murphy, R.W., Cook, J., Jia, L.Y., Huang, D.W., 2012. Wolbachia infection and dramatic intraspecific mitochondrial DNA divergence in a fig wasp. Evolution 66, 1907-1916.

Yang, L. Y., C. A. Machado, X. D. Dang, Y. Q. Peng, D. R. Yang, D. Y. Zhang, and W. J. Liao. 2015. The incidence and pattern of copollinator diversification in dioecious and monoecious figs. Evolution 69:294-304.
